# A pilot study of combined optical coherence tomography and diffusion tensor imaging method for evaluating microstructural change in the visual pathway of pituitary adenoma patients

**DOI:** 10.1186/s12886-022-02320-2

**Published:** 2022-03-12

**Authors:** Yanhua Pang, Zhi Tan, Wei Mo, Xinxin Chen, Jinfen Wei, Qing Guo, Qin Zhong, Jingxiang Zhong

**Affiliations:** 1 Department of Ophthalmology, The First Affiliated Hospital of Jinan University, 613 West Huangpu Avenue, Tianhe District, Guangzhou, 510000 Guangdong Province China; 2 Department of Ophthalmology, Affiliated Hospital of Guangdong Medical University, Zhanjiang, Guangdong Province China; 3 Department of Radiology, Affiliated Hospital of Guangdong Medical University, Zhanjiang City, Guangdong Province China; 4 Department of Neurosurgery, Affiliated Hospital of Guangdong Medical University, Zhanjiang City, Guangdong Province China; 5 Teaching and Research Center of Medical Communication Science, Affiliated Hospital of Guangdong Medical University, Zhanjiang, Guangdong Province China; 6 Department of Ophthalmology, The Sixth Affiliated Hospital of Jinan University, Dongguan, Guangdong Province China

**Keywords:** Diffusion tensor imaging, Pituitary adenoma, Optical coherence tomography, Visual pathway, Fractional anisotropy

## Abstract

**Background:**

RNFL thickness measured by optical coherence tomography (OCT) and visual pathway measured by diffusion tensor imaging (DTI) can be used to predict visual field recovery, respectively. However, the relationship between RNFL thickness and visual pathway injury in patients with pituitary adenoma (PA) remains unclear. This study aims to evaluate the combining DTI and OCT methods in observing the microstructural change in the visual pathway in patients with PA.

**Methods:**

Twenty-nine patients who were diagnosed with PA were included in the study group, and 29 healthy subjects were included as the control group. OCT detected the thickness of circumpapillary retinal nerve fiber layer (CP-RNFL) and ganglion cell layer (GCL). DTI measured the values of fractional anisotropy (FA) and apparent diffusion coefficient (ADC). Correlation between CP-RNFL and GCL thickness and FA and ADC values was analyzed in the study group.

**Results:**

Compared with the control group, the FA values of the bilateral optic nerve, chiasma, bilateral optic tract, and left optic radiation in the study group were reduced, and the ADC values of the bilateral optic nerve and optic chiasma were increased. Correlation analysis showed that the FA value of the optic chiasma was positively correlated with the average thickness of RNFL, the CP-RNFL thickness in the nasal and temporal retinal quadrants in both eyes, as well as the thickness of macular ring GCL in the nasal, supra, and inferior quadrants. The FA values of the optic nerve, optic chiasma, optic tract, and optic radiation were positively correlated with CP-RNFL thickness in the nasal and temporal quadrants.

**Conclusion:**

Combined DTI and OCT can provide a comprehensive understanding of the microscopic changes in the structure and function of the whole visual pathway in patients with PA.

## Background

Pituitary adenoma (PA) is the most common intracranial tumor, accounting for about 15% of all intracranial tumors [1]. This slow-growing benign tumor often compresses the chiasma, leading to progressive vision loss in one or both eyes, resulting in reduced vision or visual field defect [2]. Transnasal surgical treatment with optical surgical excision improves visual acuity and is the treatment of choice for most patients [3, 4]. Preoperative evaluation of the structural integrity of visual pathways is critical [5] to predict postoperative recovery of visual function. Optical coherence tomography (OCT), a widely used technique to study optic neuroretinal diseases, is regarded as the primary method to evaluate compression optic neuropathy via determining nerve fiber layer (RNFL) thickness [6]. However, the use of RNFL thickness alone in predicting visual function after pituitary tumor surgery is controversial. Yoneoka et al. suggested that regular RNFL thickness was an independent predictor of postoperative visual field recovery [7]. Zhang et al. found that PA patients with regular RNFL thickness had a combined odds ratio of 15.61 (95%CI: 4.09–59.61) in vision recovery compared with those with thinned RNFL [8]. In contrast, a study by Póczoš et al. suggested that preoperative RNFL thinning could not predict poor postoperative visual function recovery [9]. The reason for this controversy may be that the results obtained simply by RNFL changes may not be comprehensive enough to evaluate the whole optic pathway damaged by PA, especially to distinguish the microstructural damage.

Diffusion tensor imaging (DTI) is a noninvasive imaging method to exhibit the continuity and integrity of tissue structure of living tissue based on two parameters fractional anisotropy (FA) and apparent diffusion coefficient (ADC) [10]. DTI’s ability to in vivo anatomical evaluation of injury-dysfunction relationships [11, 12] makes it successful to assess PA-induced visual pathway damage [13] by reflecting the microstructural changes of the optic nerve, optic chiasma, optic tract, and optic radiation. In detail, the parameter FA (ranged from 0 to 1) reflects the anisotropic movement of water molecules within tissues in white matter fibers representing the integrity of microstructures [11]. The parameter ADC (high value represents higher diffusivity [14]) reflects the overall molecular water diffusion rate representing the state of tissue integrity at a fairly global level [15]. The multi-mode use of DTI and optical OCT is a benefit for the evaluation of the entire afferent visual system objectively [16]. However, there is still no report on the use of combining OCT and DTI techniques for the evaluation of visual pathway function in PA patients. In the present study, OCT was used to detect the thickness of RNFL, and DTI was used to detect the FA and ADC values of the optic nerve, optic chiasm, optic tract, and optic radiation in PA patients. The correlation between RNFL thickness and DTI indexes was analyzed to evaluate the damage of the whole visual pathway.

## Materials and methods

### Study design

Patients who were first diagnosed as PA in the Affiliated Hospital of Guangdong Medical University from March 2020 to July 2021 were enrolled in this study. All subjects underwent the best-corrected visual acuity, visual field, optic disc and macular OCT, and optic circuit DTI examinations. All the subjects had signed informed consent.

Inclusion and exclusion criteria for the study group: pituitary tumors confirmed by imaging magnetic resonance imaging; pituitary tumors were removed by complete sphenoid sinus resection by the endoscope and verified by pathological examination; more than 18 years old; non-contact intraocular pressure ≤ 21 mmHg (1 mmHg = 0.133 kPa); no history of intracranial disease, trauma and or intracranial surgery; no history of eye trauma, glaucoma, and no history of neuroretinal diseases, and ocular surgery; refractive error < ±6.0D (spherical lens) and < 3.00 D (cylindrical lens).

Inclusion and exclusion criteria for the control group: non-contact intraocular pressure ≤ 21 mmHg; vision or corrected vision ≥0.6, refractive errors <±6.0D (spherical lens) and < 3.00D (cylindrical lens); no history of intracranial disease and trauma, and intracranial surgery; no history of eye trauma, glaucoma, neuroretinal diseases, and no history of internal eye surgery.

### Visual field examination

The mean deviation (MD) value is a parameter used to evaluate the overall visual field defect. All patients underwent visual field examination after correction of refractive error before surgery (KowaAP7000 precision visual field meter, Japan). Visual field assessment center 30 degrees. If solid vision disappears and false negative or false positive errors exceed 20%, the test was considered unreliable and repeated. To ensure the reliability of the test results, all patients received two reliable visual field examinations.

### Magnetic resonance examination of the tumor

All patients underwent an enhanced MRI plain scan (Discovery MR750 3.0 t, GE, USA) to measure tumor size before surgery. The maximum anterior and posterior diameter, horizontal diameter, vertical diameter, and tumor height values of the suprasellar extension were measured and recorded. Each value of indexes was measured three times and averaged.

### OCT examination for RNFL thickness

The optic disc and macular area were scanned by 3D-OCT to determine the thickness of RNFL (Heidelberg Engineering Spectralis, German). RNFL indexes include the average thickness of circumpapillary RNFL (CP-RNFL), and each thickness of CP-RNFL in the nasal, supra, temporal, and inferior retinal quadrants. Macular ganglion cell layer thickness is detected in the innermost macula (diameter = 1 mm), inner ring (diameter 1-3 mm from the central fovea), and nasal, supra, bitemporal, and inferior retinal quadrants. The measured value is calculated by the software of the OCT device.

### DTI examination of FA and ADC values

T1WI and DTI scan was operated with 16-channel phased-array coil (GE3.0 T Optima MR360 imaging system). Scanning parameters for T1WI are TR/TE 12.3/5.1 ms, 256 × 256 matrix, FOV240mm × 240 mm, 1.4 mm thickness, 0 mm interval, and NEX 1 with Ax 3D BRAVO sequence. DTI scan used single excitation DW-SE-EPI sequence and parameters were set up on TR/TE 9000/100.1 ms, 128 × 128 matrix, FOV240mm × 240 mm, 1 collection, 25 diffusion sensitive gradient directions, b value =1000s/mm2, layer thickness and layer spacing of 2/0 mm, axial scanning. The scanning results were color-coded tensor FA and ADC imaging.

### Data processing for DTI

**Fig. 1:**
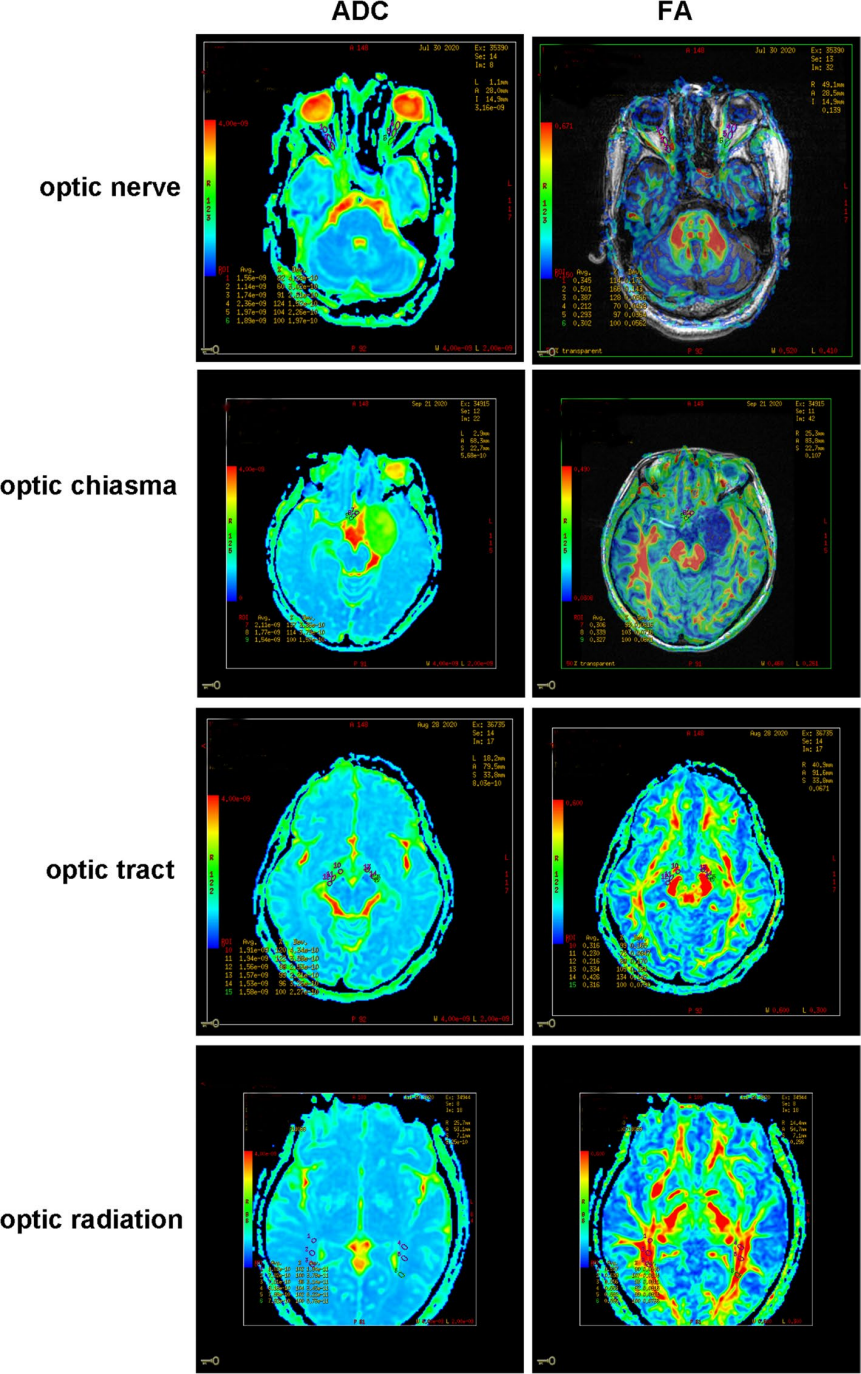
Represented image of values ADC and FA detected by OCT from four patients

Color-coded tensor FA and ADC images are set as green in the front and back direction, red in the left and right direction, and blue in the top and bottom direction. In each group, the optic nerve, optic tract, anterior, middle, and posterior parts of optic radiation, and the left, middle and right parts of optic chiasma were measured and analyzed by the software (ADW 4.2 Function Tool) of the GE3.0 NMR machine. Three regions of (ROI) were selected from the most displayed positions of the bilateral optic nerve, optic chiasma, bilateral optic tract, and radiographic images, and FA and ADC values were measured for this range. The classification and measurement method of ROI was established by referring to the classical neuroanatomical description and relevant literature [17], and the ROI size was 8 ~ 12 mm2. The FA value and ADC value of optic nerve, optic chiasma, optic tract, and optic radiation were calculated as the average of the three ROI. All data processes were operated by the same physician. The FA and ADC images are shown in Fig. 1.

### Statistical analysis

Data were analyzed using IBM SPSS24.0 software and presented as mean ± standard deviation when the data fit a normal distribution. An Independent-sample t-test was used for comparison between groups. Spearman correlation was used to evaluate the relationship between the RNFL and GCL thinning and the FA and ADC values. *P* less than 0.05 considerable statistical significance.

## Results

### Baseline characteristic of the two groups

A total of 29 PA patients were enrolled in the study, including 12 males and 17 females, aged 31–79 years, with an average of 53.655 ± 15.22 years old. The course of disease ranged from 1 to 120 months, with an average of 28.285 ± 38.49 months. Among patients, 26 cases were non-functional PA, 2 cases were pituitary growth hormone adenomas, and 1 case was pituitary prolactinocyte adenoma. The controls were 13 males and 16 females, aged 31–79 years, with an average of 50.827 ± 15.01 years. There was no significant difference in sex and age between PA patients and the controls (Table 1). The MD value of the right eye was 4.486 ± 5.846 dB, and that of the left eye was 4.086 ± 5.719 dB. The mean best-corrected visual acuity (BCVA) was 0.427 ± 0.773 logMAR in the right eye and 0.427 ± 0.664 logMAR in the left eye. MRI examination showed that the vertical diameter of the tumor in the study group ranged from 0.70 to 5.49 cm, with an average of 2.764 ± 1.165 cm. The epitaxial height of suprasellar extension ranged from 0.00 to 3.02 cm, with an average of 0.949 ± 0.913 cm. The horizontal diameter was 0.70 to 3.77 cm, with an average of 2.507 ± 0.961 cm. The vertical diameter was 0.80 ~ 4.74 cm, with an average of 2.441 ± 1.123 cm.

**Table 1:** Baseline characteristic of the two groups

	Study group (*n* = 29)	Control group (*n* = 29)	*X*^2^	*P value*
Age (years)	53.655 ± 15.06	50.827 ± 15.01		0.477
Sex				
Male	12	13	0.07	>0.05
Female	17	16		

### OCT measurement suggested RNFL and GCL thickness were thin in PA patients

Compared with the control group, the mean CP-RNFL thickness and nasal, supra, and temporal RNFL thickness in the study group were significantly thinning (*P* = 0.010, 0.039, 0.036, 0.000). There was no significant difference in the thickness of inferior RNFL between the two groups (Table 2). Compared with the control group, the nasal, supra, and inferior GCL thickness of the macular ring in the study group were significantly thinning (*P* = 0.000, 0.000, 0.000) (Table 3).

**Table 2:** Comparison of CP-RNFL thickness between the study group and control group (μm)

	Average CP-RNFL	Nasal CP-RNFL	Supra CP-RNFL	Temporal CP-RNFL	Inferior CP-RNFL
Study group (*n* = 58)	96.916 ± 23.58	67.033 ± 23.69	120.716 ± 35.17	68.650 ± 21.57	128.033 ± 32.31
Control group (*n* = 58)	108.529 ± 13.78	76.970 ± 18.79	132.479 ± 18.06	87.176 ± 24.14	136.352 ± 21.73
*P*	0.010	0.039	0.036	0.000	0.184

**Table 3:** Comparison of macular GCL thickness between the study group and control group (μm)

	Center (1 mm diameter)	Nasal internal ring	Supra internal ring	Temporal internal ring	Inferior internal ring
Study group (*n* = 58)	23.483 ± 8.79	18.112 ± 15.93	43.983 ± 11.85	45.322 ± 11.88	41.129 ± 10.27
Control group (*n* = 58)	25.416 ± 4.16	50.333 ± 5.95	52.833 ± 7.37	46.305 ± 7.59	50.555 ± 7.02
*P*	0.145	0.000	0.000	0.657	0.000

### DTI showed decreased FA values and increased ADC values in PA patients

Compared with the control group, the FA values of the bilateral optic nerve, optic chiasma, bilateral optic tract, and left optic radiation decreased (*P* = 0.025, 0.013, 0.000, 0.002, 0.004, 0.008) (Table 4), while the ADC values of the bilateral optic nerve and optic chiasma increased in the study group (*P* = 0.034, 0.004, 0.009) (Table 5).

**Table 4:** Comparison of DTI FA value between the study group and control group

	Right optic nerve	Left optic nerve	Optic chiasma	Right optic tract	Left optic tract	Right optic emission	Left optic emission
Study group (*n* = 29)	0.432 ± 0.11	0.420 ± 0.08	0.236 ± 0.10	0.369 ± 0.12	0.377 ± 0.13	0.481 ± 0.06	0.465 ± 0.08
Control group (*n* = 29)	0.493 ± 0.08	0.483 ± 0.10	0.339 ± 0.04	0.464 ± 0.09	0.471 ± 0.09	0.504 ± 0.05	0.519 ± 0.06
*P*	0.025	0.013	0.000	0.002	0.004	0.158	0.008

*FA* fractional anisotropy, *ADC* apparent diffusion coefficient, *DTI* diffusion tensor imaging

**Table 5:** Comparison of DTI ADC value between the study group and control group

	Right optic nerve	Left optic nerve	Optic chiasma	Right optic tract	Left optic tract	Right optic emission	Left optic emission
Study group (*n* = 29)	1.641 ± 0.34	1.598 ± 0.38	1.805 ± 0.29	1.261 ± 0.38	1.179 ± 0.34	0.866 ± 0.06	0.872 ± 0.07
Control group (*n* = 29)	1.463 ± 0.27	1.332 ± 0.27	1.499 ± 0.56	1.207 ± 0.30	1.260 ± 0.35	0.870 ± 0.06	0.850 ± 0.06
*P*	0.034	0.004	0.009	0.970	0.380	0.802	0.276

*FA* fractional anisotropy, *ADC* apparent diffusion coefficient, *DTI* diffusion tensor imaging

### Correlations between visual acuity and visual field defect and DTI parameters

The correlations between visual acuity and visual field defect and DTI parameters were analyzed. BCVA logMAR was negatively correlated with optic nerve FA, optic chiasma FA and optic tract FA (*r* = − 0.516, − 0.415, − 0.442; *P* = 0.004, 0.025, 0.016), and was positively correlated with ADC values of the optic nerve and optic tract (*r* = 0.369, 0.484; *p* = 0.049, 0.008). The MD value negatively correlated with optic nerve FA, optic chiasma FA and optic tract FA (*r* = − 0.470, − 0.487, − 0.432; *P* = 0.010, 0.007, 0.019) and was positively correlated with the ADC value of optic nerve (*r* = 0.391; *P* = 0.036) (Table 6) suggesting that the FA values of the optic nerve, optic chiasma, and optic tract decreased with the increase of visual field impairment of PA patients. These data suggest that the FA values of the optic nerve, optic chiasma, and optic tract decreased with increased damage of visual acuity and visual field defect (Table 6).

**Table 6:** Correlation between BCVA, MD value, tumor diameter line and DTI parameters

	Optic nerve FA		Optic chiasma FA		Optic tract FA		Optic nerve ADC		Optic tract ADC		Optic radiation ADC	
	r	*P* value	r	*P* value	r	*P* value	r	*P* value	r	*P* value	r	*P* value
BCVA (logMAR)	**−0.516**	**0.004**	**−0.415**	**0.025**	**−0.442**	**0.016**	**0.369**	**0.049**	**0.484**	**0.008**	0.235	0.220
MD value	**−0.470**	**0.010**	**−0.487**	**0.007**	**−0.432**	**0.019**	**0.391**	**0.036**	0.331	0.080	0.308	0.104
Epitaxial height of suprasellar extension	**−0.384**	**0.040**	**−0.535**	**0.003**	**−0.371**	**0.048**	0.151	0.433	0.261	0.171	0.193	0.551
Horizontal diameter	−0.026	0.893	−0.153	0.429	−0.073	0.706	0.038	0.844	0.29	0.128	**0.551**	**0.002**
Vertical diameter	−0.285	0.134	**−0.474**	**0.009**	**−0.378**	**0.048**	0.196	0.309	0.134	0.489	**0.388**	**0.038**

### Correlation between tumor diameter and DTI parameters

The epitaxial height of suprasellar extension of the tumor was negatively correlated with FA values of the optic nerve, optic chiasma, and optic tract (*r* = − 0.384, − 0.535, − 0.371, *P* = 0.040, 0.003, 0.048), indicating the tumor height of suprasellar extension positively correlated with the degree of visual path injury. The ADC values were positively correlated with the horizontal and vertical diameter of the tumor (*r* = 0.551, 0.388; *P* = 0.002, 0.038) (Table 6), suggesting that tumor compression on the optic chiasma causes optic radiation damage.

### Correlation analysis between RNFL thickness and GCL thickness and DTI parameters

**Fig. 2:**
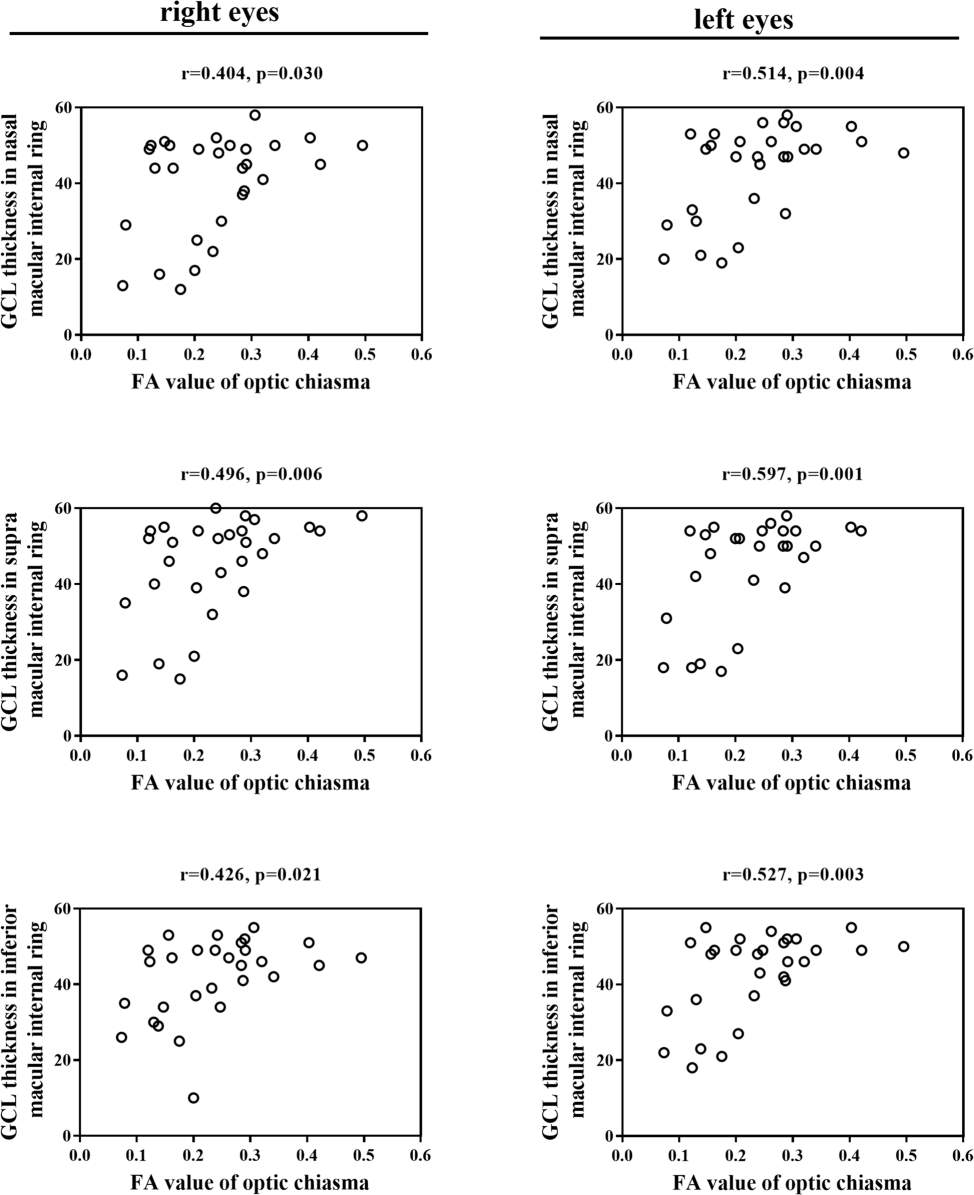
Correlation between GCL thickness and FA values of the optic chiasma

In the study group, the FA value of the optic chiasma positively correlated with the binocular average RNFL thickness, nasal and temporal CP-RNFL thickness. The correlation coefficients of these values in the right eye were r = 0.414, 0.372, 0.504 (*P* = 0.026, 0.047, 0.005) and in the left eye were *r* = 0.444, 0.436, 0.487 (*P* = 0.016, 0.008, 0.007) (Table 7). The FA value of the optic chiasma positively correlated with the nasal, supra, and inferior GCL thickness in the internal macular ring, and the correlation coefficients of these values in the right eye were *r* = 0.404, 0.496, 0.426, respectively (*P* = 0.030, 0.006, 0.021) and in the left eye were *r* = 0.514, 0.597, 0.527, respectively (*P* = 0.004, 0.001, 0.003) (Fig. 2). The FA values of the optic nerve, optic chiasma, optic bundle, and optic radiation positively correlated with the thickness of CP-RNFL in nasal and inferior retinal quadrants, and the best correlation coefficients respectively were 0.518, 0.436, 0.438, 0.474, respectively (*P* = 0.004, 0.008, 0.018, 0.009) (Table 7).

**Table 7:** Correlation between CP-RNFL thickness and DTI parameters

Versus	Average CP-RNFL	Nasal CP-RNFL	Supra CP-RNFL	Temporal CP-RNFL	Inferior CP-RNFL
Correlation coefficient of ipsilateral optic nerve FA value	0.513^*^	0.495^*^	0.410^*^	0.340	0.518^*^
*P* value	0.004	0.006	0.027	0.089	0.004
Correlation coefficient of optic chiasma FA value	0.444^*^	0.436^*^	0.398^*^	0.504^*^	0.419^*^
*P* value	0.016	0.008	0.033	0.005	0.024
Correlation coefficient of optic tract FA value	0.369^*^	0.372^*^	0.315	0.176	0.438^*^
*P* value	0.049	0.047	0.096	0.388	0.018
Correlation coefficient of optic rdition FA	0.307	0.474^*^	0.338	0.325	0.394^*^
*P* value	0.127	0.009	0.091	0.085	0.034
Correlation coefficient of ipsilateral optic nerve ADC value	−0.291	−0.403^*^	−0.340	−0.354	−0.307
*P* value	0.150	0.030	0.089	0.076	0.127
Correlation coefficient of optic rdition ADC value	−0.336	−0.379^*^	− 0.304	−0.259	− 0.206
*P* value	0.093	0.042	0.131	0.202	0.305

*FA* fractional anisotropy, *ADC* apparent diffusion coefficient, *DTI* diffusion tensor imaging, *CP-RNFL* circumpapillary retinal nerve fiber layer. *P < 0.05

## Discussion

The present study proved that OCT and DTI could evaluate the optic nerve injury caused by pituitary tumors. Moreover, the correlation between the two imaging indicators indicates that the combination of OCT and DTI methods predict the microscopic structure changes of the optic nerve.

DTI is a new non-invasive MRI technique that quantifies the diffusion direction of water molecules and is used to quantify changes in visual pathways and white matter fiber structure by measuring parameter FA and ADC [18]. Decreased FA and increased ADC value represent the loss of nerve fibers and atrophy of nerve tissue [19]. FA value is the most commonly used parameter of DTI in the study of optic nerve diseases. For example, Mu Li et al. believed that the FA value of the optic nerve was a reliable marker for evaluating glaucoma sensitivity, with a sensitivity of 79.2% and specificity of 89.6% when ≤0.412 [20]. Schmidt Ma et al. found that the FA values of the optic nerve and optic radiation in patients with glaucoma were lower than those in normal populations [21]. In patients with PA, Lilja Y suggested that the low level of FA might represent the early atrophy of the visual pathway, which was an early sign of optic nerve injury [13]. Anik et al. believe that DTI can evaluate and predict the visual recovery of patients with optic chiasma compression caused by PA after surgery and an FA value below a threshold value for a given period or an MD value higher than a preset value reflect a poor prognosis [22]. Our results showed that compared with the healthy population, PA patients had a decreased FA value and an increased ADC value in the optic pathway, indicating the loss of the optic fibers and neuronal tissue atrophy. We can see that DTI parameters exhibit the damage of the tumor to the whole optic pathway and quantify the damage degree.

RNFL of the retina is categorized into nasal chiasmatic fibers and temporal nonchiasmatic fibers in a vertical line through the fovea. The nasal RNFL mainly flowed into the nasal and temporal quadrant of the optic disc. The temporal RNFL flows into the supra and inferior quadrant of the optic disc. In our study, the thickness of nasal and temporal CP-RNFL of PA patients was thinner than that in the controls, which resulted from long-term compression of cross fibers. Although tumor compression of the optic chiasma affects the chiasmatic RNFL, it also affects the non-chiasmatic fibers [23], so the supra CP-RNFL is also thinned.

The thinning of RNFL thickness in PA is different from that in POAG due to disparate nerve damage mechanisms of PA and POAG. The pituitary adenoma directly compresses the optic chiasma, resulting in the damage of the chiasma fibers, while the optic disc nasal and temporal fibers are both chiasma fibers, thus showing thinning of the nasal and temporal RNFL. In a recent study by Felix Tonagel et al., the thickness of temporal CP-RNFL is the critical distinction between optic nerve injury of POAG and optic nerve injury caused by tumor compression. The thickness of the supratemporal and subtemporal RNFL in early or intermediate POAG is usually in the normal range or critical value, but the thickness of the temporal RNFL caused by tumor compression is usually thinner [24]. But more data are needed to clarify this phenomenon.

We found that the nasal, supra, and inferior GCL thickness of PA patients was thinner than that in the controls, which was similar to the study of Yum HR et al. [25], which suggested that the thickness of the macular ganglion cell layer and inner plexiform layer (GCIPL) in the supra and inferior of both macular area and nasal was thinner than that in the controls. Poczocnp et al. considered that the nasal and temporal GCL of PA patients with severe optic nerve compression was thinner than that of the controls before surgery [9]. These results suggest that PA patients get thinner in different quadrants of optic nerves. The reason for this may be that PA had not staged. This phenomenon needs further study.

The correlation between OCT RNFL thickness and DTI parameter is different in various diseases. Altıntas O et al. did not find any correlation between DTI parameter and RNFL thickness between amblyopia patients and normal controls [26]. Sidek et al. showed that the DTI FA value of optic nerve in glaucoma exhibits a strong correlation with the RNFL thickness [27]. Nishioka C et al. suggested that the DTI parameter closely correlated with RNFL thickness during the chronic phase of axonal degeneration (4–8 weeks) in mice with experimental autoimmune encephalomyelitis [28]. Our study for the first time found that the nasal and inferior CP-RNFL thickness of PA patients positively correlated with the FA value of the entire visual pathway. The structural composition of the optic nerve that the nasal and temporal semi-chiasmatic RNFL of the retina merged into the nasal and temporal sides of the optic disc, and the inferior RNFL were directly compressed by tumor may explain this phenomenon. When the binocular chiasmatic nerve fibers are compressed at the optic junction, the damage of entire optic pathway can be quantified by the FA value. The FA value of optic chiasma positively correlated with the CP-RNFL thickness of the temporal optic disc and the GCL thickness of the intranasal macular ring of the binocular optic disc, which is where the mamellar bundle is located. Kawaguchi T1 et al. found that the CP-RNFL thickness in the temporal quadrant significantly correlated with the recovery of visual function [29]. It is thought that the mammary bundle converges from the temporal side of the optic disc, and the thinning of the temporal nerve fiber layer has a pivotal impact on vision. Glebauskiene Bet al. found that the temporal CP-RNFL thickness of pituitary tumors with suprasellar extension was thinner than that without suprasellar extension; the thickness of temporal CP-RNFL positively correlated with the distance between the optic chiasm and the pituitary tumor [30]. Previous studies have analyzed the relationship between temporal CP-RNFL thickness and visual function from the perspective of structure. In contrast, our results from a functional perspective found a positive correlation between temporal CP-RNFL thickness and intranasal GCL thickness of the macular ring and optic chiasma function.

Our study found that visual acuity and visual field negatively correlated with FA values of the optic nerve, optic chiasma, and optic tract in PA patients, indicating that the FA values decreased with the increase of visual acuity and visual field impairment. As for the correlation between tumor size and DTI parameters, our data suggested that the epitaxial height of suprasellar extension of the tumor closely related to the optic nerve, optic chiasma, and optic tract damage compared with the anterior and posterior diameter, horizontal diameter, and vertical diameter of the tumor. This is similar to the previous study of Gan, L et al. [31] which found that tumor epitaxial height on the suprasellar extension correlated with MD value. Our study investigated the relationship between the epitaxial height of suprasellar extension and visual path function. A previous study by Danesh-Meyer et al. has pointed out that anterograde degeneration is an important mechanism causing optic radiation injury [32]. Our data suggested that ADC value correlated to the horizontal and vertical diameter of the tumor, suggesting that the tumor’s compression of optic chiasm may also damage optic radiation. Further study is needed to confirm the visual radiation damage caused by the compression of the tumor on the optic chiasma.

There are four shortcomings in this study. First, DTI is rarely used in clinical practice because of the time-consuming post-processing and high cost. Second, how to improve the three-dimensional spatial resolution of the optic path is still a challenge, because there are many cross fibers in the whole optic path, especially at the optic chiasma. Third, errors may occur when measuring DTI parameters using ROI. The length and thickness of the optic pathway have anatomical differences, and the corresponding ROI in each subject will not represent the same part of the optic pathway due to individual differences. Forth, OCT measurements do not distinguish between normal and abnormal structures. If the patient with optic nerve edema and an unclear refractive medium, retinal structure stratification will be affected and the results will be unreliable.

## Conclusions

In summary, in patients with PA, DTI can be used to measure the signal transduction of nerve fibers and OCT measures the thickness of nerve fibers. The combination of DTI and OCT detects microstructural pathological changes of the visual pathway beneficial to comprehensively understand the microscopic changes in the structure and function of the whole visual pathway. Our findings provide a useful reference for clinical treatment strategy.

## Data Availability

The material and data are available from the corresponding author Yanhua Pang (pang1049371818@163.com) on reasonable request.

## Abbreviations

DTI: Diffusion tensor imaging
OCT: Optical coherence tomography
PA: Pituitary adenoma
CP-RNFL: Circumpapillary retinal nerve fiber layer
GCL: Ganglion cell layer
FA: Fractional anisotropy
ADC: Apparent diffusion coefficient
